# Supplementary material for: A Humanized Yeast Phenomic Model of Deoxycytidine Kinase to Predict Genetic Buffering of Nucleoside Analog Cytotoxicity
Source: Genes (Basel). 2019 Sep 30;10(10):770. doi: 10.3390/genes10100770 (PMC6826991; doi:10.3390/genes10100770)
Supplement: Supplementary file 1 [file genes-10-00770-s001.zip › Additional_Files/Additional_File7_GOTermHeatmaps.zip!/Additional_File7_GOTermHeatmaps/histone_H3-K4_methylation.pdf]

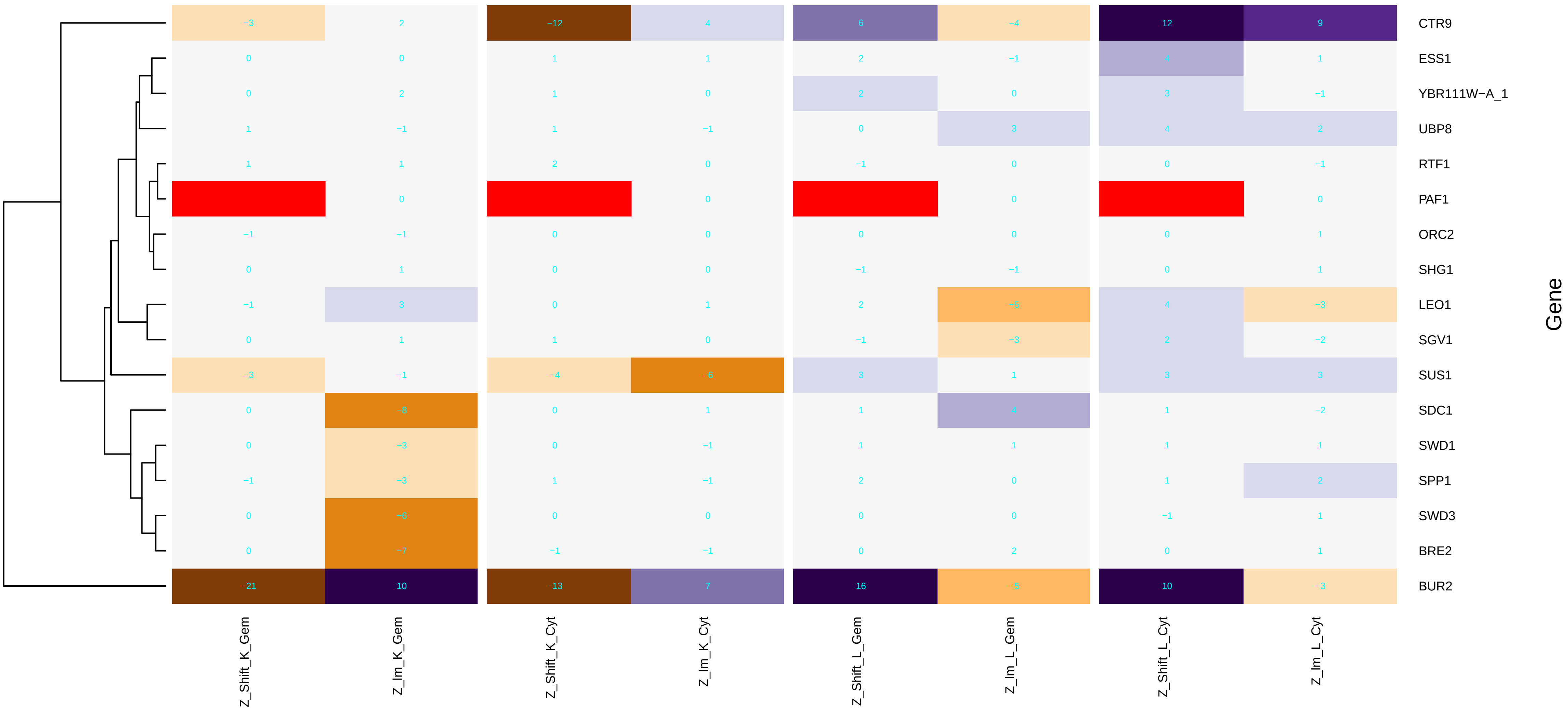
CTR9 −3 2 −12 4 6 −4 12 9
ESS1 0 0 1 1 2 −1 1
YBR111W−A_1 0 2 1 0 2 0 3 −1
UBP8 1 −1 1 −1 0 3 4 2
RTF1 1 1 2 0 −1 0 0 −1
PAF1 0 0 0 0
ORC2 −1 −1 0 0 0 0 0 1
SHG1 0 1 0 0 −1 −1 0 1
LEO1 −1 3 0 1 2 −5 4 −3
SGV1 0 1 1 0 −1 −3 2 −2
SUS1 −3 −1 −4 −6 3 1 3 3
SDC1 0 −8 0 1 1 1 −2
SWD1 0 −3 0 −1 1 1 1 1
SPP1 −1 −3 1 −1 2 0 1 2
SWD3 0 −6 0 0 0 0 −1 1
BRE2 0 −7 −1 −1 0 2 0 1
BUR2 −21 10 −13 7 16 −5 10 −3
Gene
Z_Shift_K_Gem
Z_Im_K_Gem
Z_Shift_K_Cyt
Z_Im_K_Cyt
Z_Shift_L_Gem
Z_Im_L_Gem
Z_Shift_L_Cyt
Z_Im_L_Cyt
